# The gut-retina axis: a new perspective in the prevention and treatment of diabetic retinopathy

**DOI:** 10.3389/fendo.2023.1205846

**Published:** 2023-07-04

**Authors:** Haiyan Zhang, Ya Mo

**Affiliations:** ^1^ Chengdu University of Traditional Chinese Medicine, Sichuan, China; ^2^ Affiliated Hospital of Chengdu University of Traditional Chinese Medicine, Sichuan, China

**Keywords:** diabetes mellitus (DM), diabetic retinopathy (DR), gut-retina axis, gut microbiota, retina, mechanics, treatment

## Abstract

Diabetic retinopathy (DR) is a microvascular lesion that occurs as a complication of diabetes mellitus. Many studies reveal that retinal neurodegeneration occurs early in its pathogenesis, and abnormal retinal function can occur in patients without any signs of microvascular abnormalities. The gut microbiota is a large, diverse colony of microorganisms that colonize the human intestine. Studies indicated that the gut microbiota is involved in the pathophysiological processes of DR and plays an important role in its development. On the one hand, numerous studies demonstrated the involvement of gut microbiota in retinal neurodegeneration. On the other hand, alterations in gut bacteria in RD patients can cause or exacerbate DR. The present review aims to underline the critical relationship between gut microbiota and DR. After a brief overview of the composition, function, and essential role of the gut microbiota in ocular health, and the review explores the concept of the gut-retina axis and the conditions of the gut-retina axis crosstalk. Because gut dysbiosis has been associated with DR, the review intends to determine changes in the gut microbiome in DR, the hypothesized mechanisms linking to the gut-retina axis, and its predictive potential.

## 1 Introduction

Diabetic retinopathy (DR) has emerged as a leading cause of visual impairment in working-age people in various regions (1–3). According to the International Diabetes Federation (IDF), more than 500 million people worldwide will have diabetes mellitus (DM) by 2021 (4). The presence and progression of DR are associated with a significant increase in healthcare costs. Diabetes-related direct health expenditures were USD 760 billion in 2019 and are expected to rise to USD 825 billion by 2045 (5). Numerous studies demonstrated that approximately one in every three diabetic patients has DR (6). Given the high incidence of DR and the Global Burden of Disease estimate, it is critical to investigate the predictive potential for DR progression and potential therapeutic targets.

DR has been considered a microvascular complication, a known complication of DM (7–9). Studies suggest that neurodegeneration is an early event in its pathogenesis, and abnormalities in retinal function can occur in patients with no evidence of microvascular abnormalities (10, 11). The American Diabetes Association (ADA) recently defined DR as a precise neurovascular complication (12). Although studies revealed that DR is caused by chronic hyperglycemia, with retinal neovascularization, chronic inflammation, disorders of glucolipid metabolism, and immune response as its hallmarks (13–16), the exact pathogenesis remains unknown. The intestinal microbiota is a large, diverse colony of microorganisms colonizing the human intestine. The intestinal microbiota evolved symbiotically with its host and plays an important role in regulating nutrient absorption (17) and metabolism (18–20), maintaining the intestinal mucosal barrier (21), intestinal immunity and pathogen defense (22, 23). Studies indicated that the intestinal microbiota is involved in the pathophysiological processes of DR and plays an important role in DR development (24, 25).

The gut microbiota is primarily made up of bacteria. However, it also contains other commensals such as archaea, viruses, fungi, and protists (The term “microbiota” refers to consortia of microorganisms living in a specific environment, whereas “commensals” refers to microorganisms that colonize hosts without causing disease) (26). Following their functions, the intestinal microbiota can be classified as commensal, probiotic, or pernicious bacteria. The primary role of probiotics is to improve nutrient digestion and absorption, regulate lipid metabolism, and reduce the inflammatory response (27–29). Simultaneously, pernicious bacteria can activate the inflammatory response *in vivo*, disrupt the function of the intestinal epithelial barrier, and cause metabolic disorders (30, 31). Dysbiosis of the gut microbiota, also known as gut dysbiosis, is primarily characterized by a reduction in the diversity and abundance of bacteria and fungi, particularly those associated with dysfunction and various pathologies (32).

Moreover, dysbiosis of the gut microbiota can result in various gastrointestinal diseases and processes in distal tissues other than the intestine, such as joints, mucous membranes, and the eyes, which are common sites of invasion (33–35).In addition, new molecular biology-based techniques enable the identification and quantification of microbiota by analyzing DNA and RNA extracted from fecal samples. The studies described in the preceding sections support the notion that gut microbiota has become a hotspot for disease research.

Since Rowan (36) et al. introduced the concept of the “gut-retina axis” and demonstrated the existence of gut-retina crosstalk, the significance of gut microbes as important modulators of ocular disease have grown (37). Scientists identified that diet, probiotics, and antibiotics could influence the gut microbiota and, thus, the development of retinal disease (38). Increased intraocular pressure, glucose accumulation in vessels, and neovascularization can affect the health of the eye in poorly controlled diabetes (39). These processes are associated with microvascular complications in the eye, such as cataracts, glaucoma, and DR (40). DR, a complication of poorly controlled diabetes, can eventually lead to blindness (41). Dysbiosis of the gut microbiota is closely linked to the occurrence, development, and prognosis of DR.

On the one hand, an increasing number of studies have demonstrated that gut microbiota plays a role in retinal neurodegeneration (42, 43), in retinal inflammatory processes (44), and affect glucose metabolism, insulin resistance, and entero-insulin secretion (45). Conversely, alterations in the gut bacterial microbiome in people with RD, and thus dysbiosis of gut microbiota, can also cause or aggravate DR (46). For example, carnosine was depleted in DR patients compared to healthy controls. Carnosine is an endogenous dipeptide composed of β-alanine and L-histidine with significant antioxidant and anti-inflammatory properties (47). These findings suggest that gut microbiota mediates gut-retinal communication, which is important in DR.

The present review aims to highlight the importance of gut microbiota in DR. The critical role of gut dysbiosis in the development and progression of DR is discussed briefly. Subsequently, the concept of the gut-retina axis and the mediators and conditions that allow gut-retina crosstalk will be investigated, focusing on the mechanisms involved in regulating DR by the intestinal microbiota. Finally, diet and antibiotics strategies for treating DR via the regulated intestinal microbiota and, thus, the treatment of DR will be described.

## 2 Microbiota and ocular diseases

### 2.1 Gut microbiota and ocular diseases

Recent studies have confirmed the presence of many microorganisms, such as bacteria, viruses, and fungi, on the human body surface and within the body (48). These microorganisms are ten times higher than the body’s cells and have 100 times more genes than the body’s genome, with 1000 to 1150 bacteria colonizing the intestine (49, 50). Although many microorganisms exist in the human gut, only about 160 species belong to the advantage bacterium group (51). The human gut microbiota is primarily composed of two dominant bacterial phyla (human microorganisms are classified by phylum, order, family, genus, and species), *Firmicutes* and *Bacteroidetes*, which account for more than 90% of the entire community, and other subdominant phyla such as *Proteobacteria*, *Aspergillus*, *Actinomycetes*, and *Clostridium* (52). Different intestinal flora interacts in the intestinal micro-ecosystem, and the intestinal flora and their host have a mutually beneficial commensal relationship (53, 54). It maintains a complex dynamic balance in healthy populations that can help the body with various physiological functions, mainly limiting the colonization of pathogenic intestinal bacteria and maintaining the integrity of the intestinal epithelial barrier and immune homeostasis (55, 56). In addition, the intestinal flora decomposes and utilizes food residues to provide humans with essential vitamins, amino acids, and other nutrients through the mediation of a series of digestive enzymes. It can also metabolize harmful substances like nitrosamines and lactic acid. Therefore, intestinal microorganisms play an important role in the human micro-ecosystem. When there is gut dysbiosis, the intestinal micro-ecosystem is disrupted, resulting in chronic inflammatory responses and immune diseases in the eye (**Table 1**), such as fungal keratitis (61), DR (62), age-related macular degeneration (AMD) (63), and uveitis (UVT) (64). In addition, there is a link between inflammatory bowel diseases and ocular diseases; 10% of subjects with inflammatory bowel disease have ocular diseases (such as episcleritis, uveitis, and conjunctivitis) (65). In humans, patients with DR have a significantly lower proportion of *Bacteroidetes* and *Actinobacteria* than healthy individuals (45, 46).

**Table 1:** Alterations of bacteria in the fecal microbiota of patients with ocular diseases.

Study (Author, (Publication Year)	Ocular Diseases	Study design	Major Results	Conclusion	
**Healthy controls (HC) and Subjects with uveitis (UVT) were compared.**			1. It revealed reduced diversity of several anti-inflammatory organisms, including *Faecalibacterium, Bacteroides*, *Lachnospira*, *Ruminococcus*, and members of *Lachnospiraceae* and *Ruminococcaceae* families, while enrichment of *Prevotella* (pro-inflammatory) and *Streptococcus* (pathogenic) OTUs in UVT microbiomes compared to HC. 2. Decrease in probiotic and antibacterial organisms was observed in UVT compared to HC microbiomes.	The study demonstrates dysbiosis in the gut bacterial communities of UVT patients in an Indian cohort.
Kalyana et al. (57) (2018),	UVT	Randomized (HC, n = 13; UVT, n = 13), Indian cohort		
**Cases with Dry Eye and controls were compared.**			Among cases, 27 were relatively more abundant, including ten *Lactobacillus* and four *Bifidobacterium* species. A relative depletion of five species was identified in patients compared with controls, notably *Fusobacterium* varium and *Prevotella stercorea.*	Differences in gut microbiome composition were found in individuals with Dry Eye compared with controls.
Goodman et al. (58) (2022),	Dry Eye	Case-Control Study (Cases, n = 13; Controls, n = 13)		
**Patients with AMD and controls were compared.**			1. The genera *Anaerotruncus*, Oscillibacter, Ruminococcus torques, and *Eubacterium ventriosum* were relatively enriched in patients with AMD, whereas *Bacteroides eggerthii* was enriched in controls. 2. Patient’s intestinal microbiomes were enriched in genes of L-alanine fermentation, glutamate degradation, and arginine biosynthesis pathways and decreased in fatty acid elongation pathway genes.	Modifications in the intestinal microbiome are associated with AMD,
Zinkernagel et al. (59) (2017),	AMD	Randomized (Patients with AMD, n = 12; controls, n = 11), Participants were recruited from the Department of Ophthalmology, University Hospital Bern (Inselspital), Switzerland.		
**The healthy controls (HC) and fungal keratitis (FK) patients were compared.**			*Faecalibacterium prausnitzii*, an anti-inflammatory bacterium, and *Megasphaera*, *Mitsuokella multacida*, and *Lachnospira* are butyrate producers enriched in HC. In contrast, *Treponema* and *Bacteroides fragilis*, which are pathogenic, were abundant in FK patients.	1. The distinct patterns of gut bacterial composition in FK and HC samples. 2. Dysbiosis in the gut bacterial microbiomes of FK patients compared to HC
Kalyana et al. (60) (2018),	fungal Keratitis	Randomized (HC, n = 31; FK, n = 32, The participants were recruited from the southern part of India.		
**The healthy controls (HC, n = 30), people with T2DM without DR (n = 25), and people with T2DM and DR (n = 28) were compared**			The microbiomes of people with T2DM and DR were significantly different. Both DM and DR microbiomes revealed a decrease in anti-inflammatory, probiotics, and other bacteria that could be pathogenic compared to HC, and the observed change was more pronounced in people with DR.	Dysbiosis in the gut microbiomes, at phyla and genera levels, was observed in people with T2DM and DR compared to HC. People with DR exhibited more significant discrimination from HC.
Das et al. (46) (2021),	DR	Randomized, subjects were recruited from South India		

UVT, Uveitis; AMD, Age-related macular degeneration; T2DM, Type 2 diabetes; DR, Diabetic retinopathy.

### 2.2 Ocular surface microbiota in patients with DR

Several studies have used traditional microbial cultures and 16S rRNA gene sequencing to describe the commensal microbiota on the ocular surface. Under normal physiological conditions, the microbiota is relatively stable, with low diversity and abundance, while still playing an important role in maintaining ocular surface homeostasis (66, 67). However, Suwajanakorn et al. (68) used next-generation sequencing analysis to demonstrate the importance of DR and glycemic control status in influencing changes in the ocular surface microbiome. Subsequent studies identified that microbes could be transferred to the retina of type 1 diabetic mice with retinopathy through gut and plasma microbiota (69). Furthermore, the microbiota composition varies throughout the body, including the eye. Although the overall gut microbiota comprises *Firmicutes* and *Bacteroidetes* (70), the ocular surface microbiota primarily comprises *Proteobacteria* and *Actinobacteria* (71, 72). *Proteobacteria*, *Actinobacteria*, and *Firmicutes* account for over 87% of all microorganisms present in the eye (73). With further investigation, the doctrine that active microbiota is present in the eye has been broken. For example, the internal eye compartment is sterile, Whereas the external compartment is exposed to environmental microorganisms (74).

### 2.3 Prevalence of gut dysbiosis in DR patients

As a metabolic disease caused by multiple factors, the gut microbiota composition differs between T2DM patients and healthy individuals. For example, significant reductions in the proportion of *Firmicutes* and *Clostridium* appear in the microbiota of diabetic patients compared to healthy controls (75). Subsequent studies have confirmed the role of gut flora in systemic metabolism and T2DM. Qin et al. (76) and Karlsson et al. (77) performed metagenomic sequencing in Chinese and Swedish diabetic patients, respectively, demonstrating that T2DM was characterized by gut dysbiosis. Further research has linked dysbiosis of the gut flora to insulin resistance (IR) and abnormal lipid metabolism, which are important factors in the pathogenesis of T2DM (78). In addition, *Bacteroidetes* to *Firmicutes* ratio (B/F ratio) is a potential diagnostic biomarker for DM (79, 80).

Previous studies have demonstrated that the pathogenesis of T2DM is commonly associated with altered gut microbiota. However, it is unclear whether diabetic patients with or without retinopathy have different gut microbial dysbiosis. Scientists investigated this and identified that DR patients have intestinal dysbiosis similar to T2DM patients, with the main differences being a decrease in microbial diversity (81), changes in microbial composition and structure (37, 46), low levels of beneficial microflora and higher levels of pathogenic bacteria (46, 82). Huang et al. (83) found increased *Bifidobacterium* and *Lactobacillus* levels and decreased *Escherichia-Shigella*, *Faecalibacterium*, *Eubacterium_hallii*_group, and *Clostridium* genera in DM and DR patients compared to the healthy population. Furthermore, patients with DR have a different gut microbiota than those with diabetes, but little variability exists among them. Moreover, Prasad et al. examined the retinas of diabetic mice and determined that gut microbial dysbiosis aggravated retinal impairment and inflammation (69). All these studies confirm that dysbiotic gut microbiota characterized DM and DR.

## 3 Overview of gut-retina axis

For decades, scientists have studied the relationship between the gastrointestinal (GI) tract and the brain, and numerous studies have confirmed the existence of the brain-gut axis. The “gut-brain axis” refers to the specific linkage between the GI tract and the central nervous system (CNS), which consists of a bidirectional exchange between the two (84). The presence of the brain-gut axis suggests that CNS regulates and governed gut metabolic activity, and there is growing evidence that not only the brain (CNS) can influence GI tract function, but the gut flora can also influence the development of CNS diseases (85, 86). For example, several studies demonstrated that amyloid deposits and neuronal fiber tangle deposits in the enteric nervous system (ENS) of patients with Alzheimer’s disease are similar to those found in the brain parenchyma (87, 88). Lewy vesicles, which appear in the brain of patients with Parkinson’s disease, have also been identified in enteric neurons (89).

The possibility of an interconnection between the eye and CNS has long been debated because the retina is an extension of the brain (90). The retina is the light-sensitive neural tissue that lines the back of the eye. In anatomical and developmental terms, the retina is a brain extension known as the ‘peripheral brain.’ Both organs consist of neurons derived from a neural tube with a multilayered cellular structure and synaptic connections. Moreover, the retina transmits information to the brain’s visual cortex via the optic nerve, which converts optical signals into nerve impulses. In addition, the retina has the advantages of clear structural stratification, visualization, ease of observation, and relative ease of functional testing compared to the brain, making it an ideal model for observing and studying neurological diseases (91).

Many features of neurodegenerative processes in the CNS are similar to those observed in the retina, and some CNS neurodegenerative diseases can affect the retina and vice versa (92). Retinal lesions, such as ganglion cell layer thinning, can occur early in Alzheimer’s disease (93). Retinal chronic progressive neurodegeneration, which can happen in the elderly, can result in eye disorders like glaucoma, AMD, and DR (94). Therefore, scholars have questioned whether the concept of a brain-gut axis applies to the retina independently of the brain, that is, whether the gut-retina axis can be distinguished from the gut-brain axis (64).

Subsequent studies have confirmed the existence of the gut-retina axis and demonstrated that dysregulation of the gut microbiota contributes to the development of ocular diseases (95–97). Moreover, the concept of “gut-retina axis” was formally proposed (98, 99), demonstrating that the gut-retina axis is closely related to ocular immune system homeostasis and plays an important role in various ocular diseases, such as AMD (36, 63), UVT (100), and glaucoma (101). The intestinal-ocular axis has emerged as a new area of basic and clinical research in ophthalmology. However, more in-depth research is needed to confirm and support the existence of the gut-retinal axis.

## 4 How the intestinal microbiota achieves mutual communication between the gut-retina

The gut-retina axis is an emerging concept that describes a strong interaction between the gut host-microbiota interface and the retina. Because the retina is immune-privileged, a critical question is how this gut-retina crosstalk can be validated. The retina is a ten-layer complex composed of numerous cells, including glial cells (Müller cells, astrocytes, and microglia), retinal microvascular endothelial cells (RMECs), retinal pigment epithelium (RPE), and all types of neurons (102, 103). RPEs, RMECs, and tight junctures form the outer and inner blood-retinal barrier (BRB). The integrity of the BRB is crucial for the function of various cells within the retina because it prevents the entry of peripheral pathogens, pathogen-associated molecular patterns (PAMPs), and leukocytes, rendering the retina an immune-privileged tissue (104). Therefore, scientists have conducted numerous studies that have revealed that the crosstalk between the gut and retina is primarily achieved through the following pathways.

The interaction between microbes, gut-derived products, and the retina can be explained by the disruption of the BRB, which is common in retinal diseases (105, 106). The GI epithelium serves as a broad interface with the external environment. Single epithelial cells, also called intestinal cells, are tightly connected and cover the inner surface of our intestinal mucosa. These cells provide a barrier by using transcellular and paracellular transport mechanisms to selectively regulate the exchanges of luminal toxins, antigens, nutrients, and water absorption between the inner and outer environments (107). Conversely, the GI epithelial barrier must maintain rapid cell renewal and barrier integrity while being exposed to continuous environmental assaults. Dysbiosis of the intestinal microbiota and inflammatory response in the presence of specific eye diseases (such as DR and UVT) can lead to intestinal barrier impairment, which increases permeability (43). Consequently, impaired gut barrier function leads to the excessive translocation of gut-derived products (such as LTA, PGN, and LPS) and even live gut bacteria into the bloodstream (108). A recent study found microbiota in the intraocular environment of healthy populations and patients with ocular diseases, breaching the dogma that the intraocular environment is sterile (109).

Short-chain fatty acids (SCFAs) are beneficial microbial metabolites produced only in the gut. Chen et al. (110) demonstrate that SCFAs can cross the BRB via systemic circulation and reach the retina, triggering an innate immune response. Consistent with this, data indicate that increased intestinal permeability caused by altered gut microbiota may allow for more significant translocation of gut metabolites and products, which may modulate retina-specific immune cells (111). In addition, studies have confirmed that SCFAs entering the systemic circulation are transported via the monocarboxylate transporter (MCT-1) across the blood-brain barrier and function in the CNS (112). SCFAs may be able to enter the retina and exert regulatory effects because MCT-1 is also present in BRB (113). All these studies suggest the presence of intraocular crosstalk.

## 5 Mechanism of gut-retina axis regulation in DR

**Figure 1:**
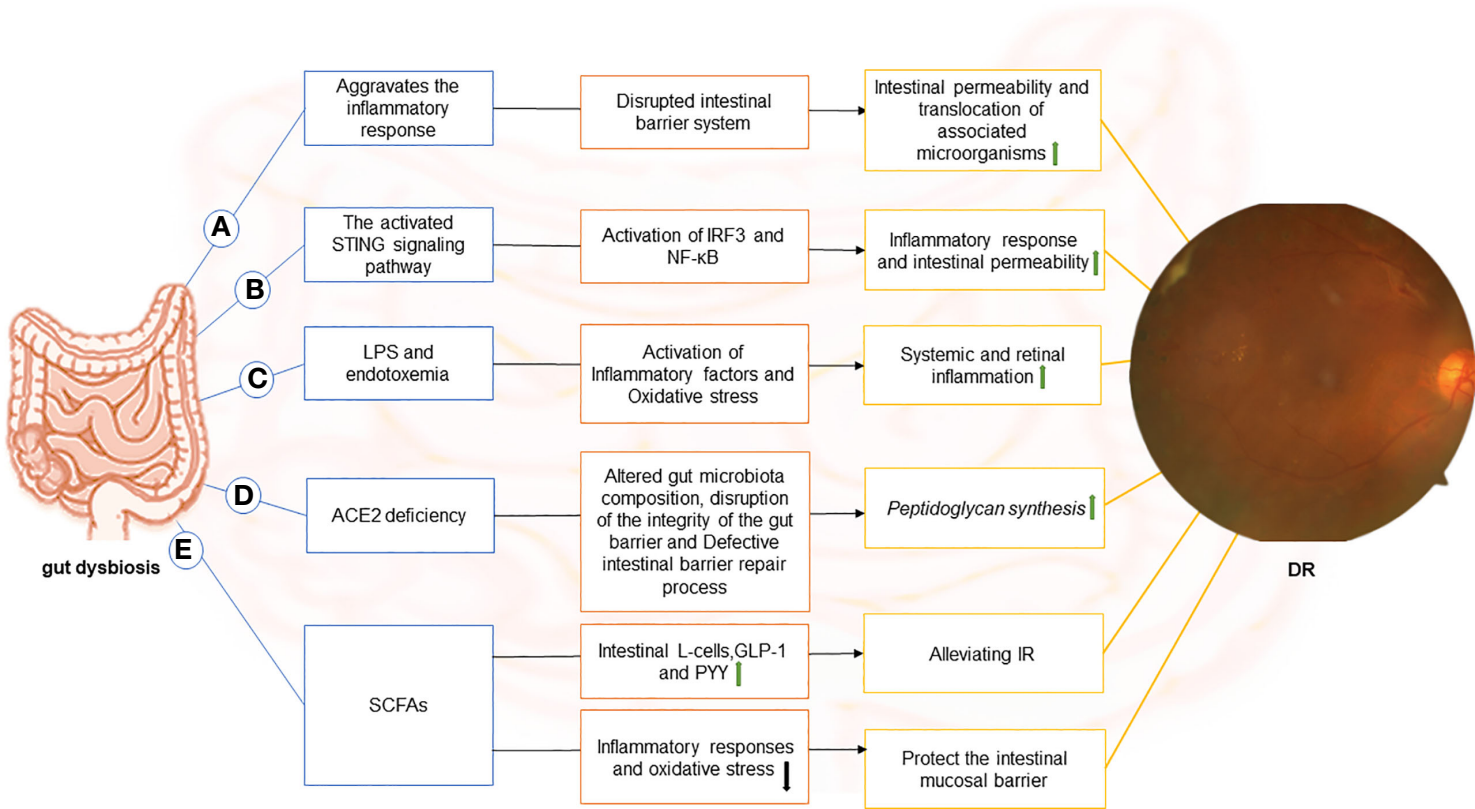
The image depicts the various possible mechanisms that connect the gut to the retina in DR and the components of each hypothetical gut-retina axis. **(A)** dysbiosis of the gut microbiota in diabetic patients causes local (including gut) and systemic inflammation. Subsequently, with inflammation, the intestinal barrier system is compromised. In this context, intestinal permeability increases, and associated microorganisms translocate. **(B)** stimulator of interferon genes (STING) pathway-mediated inflammatory signaling is activated, leading to activation of interferon regulatory factor 3 (IRF3) and nuclear factor-κB (NF-κB), which exacerbates intestinal barrier disruption. In this case, gut microbial products translocate into the blood and reach the retina through the damaged blood-retinal barrier, causing chronic activation of the STING pathway in the retina and contributing to the progression of DR disease. **(C)** endotoxemia can result from the release of lipopolysaccharide (LPS) in the gut and its entry into the blood. LPS can diffuse directly into the circulatory system and promote the release of inflammatory factors through receptor-ligand binding (primarily to CD14 and TLR4) in DM due to increased intestinal permeability or absorption via enterocytes. Moreover, binding to TLR4 increases oxidative stress, leading to systemic and retinal inflammation.**(D)** Angiotensin-converting enzyme 2 (ACE2) deficiency alters the gut microbiome composition in diabetic mice, disrupts intestinal barrier integrity, and results in intestinal barrier repair defects. The disruption of the intestinal vascular barrier causes peptidoglycan synthesis in mice, which enters the plasma and promotes DR. **(E)**Short-chain fatty acids (SCFAs) inhibit inflammatory responses and oxidative stress, suppress endotoxin-induced inflammation, and protect the intestinal mucosal barrier. It also stimulates intestinal L-cells and promotes the secretion of glucagon-like peptide-1 (GLP-1) and endocrine-regulating peptide (PYY) to alleviate IR. .

The studies described in the preceding sections support a causative role of microbiota in triggering DR, but the specific mechanisms involved remain elusive. Gut dysbiosis has been associated with DM and DR. The gut-retina axis could be a potential target for preventing DR, a well-known complication of DM. A critical question now is how the gut microbiota influences the development and treatment of DR through the gut-retina axis. We reviewed the literature and identified that the hypothesized mechanisms relating to the gut-retina axis include disruption of intestinal barrier function, activation of the stimulator of interferon genes (STING) signaling pathway, production of lipopolysaccharide (LPS), angiotensin-converting enzyme 2 (ACE2) deficiency, and affecting gut microbiota metabolites (**Figure 1**).

### 5.1 Affecting intestinal barrier system function

The intestinal mechanical and biological barriers are formed by the hierarchical and regular distribution of intrinsic intestinal bacteria in the intestinal epithelial cells, mucus on the mucosal surface, and the tight connection between cells. Both contribute to the human intestinal barrier system, which protects the organism from harmful or foreign pathogenic bacteria. The intestinal biological barrier primarily consists of *Bifidobacterium* and other bacteria found in the deep layer and *Peptostreptococcus* in the intermediate layer, which accounts for more than 99% of intestinal bacteria. These intrinsic intestinal bacteria act as a biological barrier by pre-empting colonization sites, competing for nutrients, producing organic acids and SCFAs to lower intestinal pH, producing bacterins, and inducing a moderate inflammatory response (114).

Gut microbiota is being extensively investigated for its role in DM and its complications. Changes in the gut-microbiome cause pathological inflammation and accelerate DR progression. Consequently, it influences the immune system and homeostasis locally (within the gut) and systemically (115). In this context, increased intestinal permeability and associated microbial translocation are important in the pathogenesis of DR (116). Furthermore, this contributes to the chronic systemic inflammatory process and further disrupts the intestinal barrier system. However, it has been demonstrated that even in the absence of ocular infection, the eye is susceptible to inflammatory disease, which is influenced by intraocular microbiota dysbiosis (103). In contrast, the relationship between the initiating factors of intraocular microbiota dysbiosis and DM leading to inflammation requires further investigation.

### 5.2 Stimulator of interferon genes signaling pathway-mediated inflammation

In various inflammatory diseases, aberrant regulation of the STING pathway has emerged as a critical pathogenic mechanism (117). STING is an endoplasmic reticulum (ER) adaptor protein commonly expressed in the ER. STING activation by the cytoplasmic DNA sensor cycle GMP-AMP synthase (cGAS) causes the activation of the nuclear factor-κB (NF-κB) and the transcription factor interferon regulatory factor 3 (IRF3) (118). A positive feedback loop between dysbiosis and abnormal activation of the STING pathway in the intestine is associated with increased intestinal permeability (119). There is a possibility that dysbiosis in DR patients disrupts intestinal homeostasis and aggravates barrier dysfunction through the erroneous accumulation of STING in the gut. Subsequent translocation of microbial products into the blood allows access to the retina via the impaired BRB, resulting in chronic activation of the STING pathway in the retina, contributing to disease progression (119). In addition, the STING pathway has been linked to changes in the retina and retinal cells of patients with ocular diseases (120).

### 5.3 LPS

LPS, a gut microbial-derived product composed of lipid A-based glycolipid found on the outer membrane of gram-negative bacteria, is thought to be a pro-inflammatory mediator of insulin resistance. It is challenging to shed from the outer membrane of gram-negative bacteria in healthy populations, but it becomes detached and toxic when bacteria are lysed or damaged (121). LPS is released into the gut, and when it enters the blood, it causes LPS-related toxicity, known as endotoxemia (122). LPS can enter the circulatory system by direct diffusion due to increased intestinal permeability in DR or by absorption through enterocytes. LPS is transported in the blood by lipopolysaccharide-binding proteins, binding CD14 and Toll-Like Receptor 4(TLR4) in peripheral tissues such as skeletal muscle and adipose, causing macrophage aggregation in adipose tissue, promoting the release of inflammatory factors, inducing abnormal phosphorylation of IRS-1, and leading to IR (123). When it binds to TLR4, it activates NF-κB and increases oxidative stress, leading to systemic and retinal inflammation (123).

LPS causes endotoxemia and promotes inflammation, whereas other microorganisms produce protective effects. For example, *Lactobacillus*, *Bacteroides*, *Faecalibacterium*, *Akkermansia muciniphila* (*A. muciniphila*), and *Roseburia* are known to down-regulate the pro-inflammatory cytokines in the intestine. *Bacteroides* and *A. muciniphila* improve intestinal barrier function. *Bacteroides* reduce intestinal permeability, decrease LPS production, and improve endotoxemia by up-regulating the colonic tight junction gene expression (122). *A. muciniphila* reduces intestinal permeability by regulating extracellular vesicles, which improves intestinal tight junctions by activating AMP-activated protein kinase in intestinal epithelial cells, thereby enhancing intestinal defence (124). In addition, the outer membrane protein of *A. muciniphila* up-regulates tight junction protein expression and inhibits CB-1, improving intestinal integrity and reducing LPS levels (125).

### 5.4 The gut-retina axis regulates DR via angiotensin-converting enzyme 2 and peptidoglycan

Takkar et al. (126) reported that ACE2 and peptidogly can play an important role in regulating the pathogenesis of DR. In type 1 diabetic mice, ACE2 deficiency alters gut microbiome composition and gut integrity, as well as defects in the gut barrier repair process (127). Disruption of the intestinal vascular barrier and increased growth of *Bifidobacterium animalis* contributes to peptidoglycan synthesis. Consequently, bacterial peptidoglycan enters the bloodstream and promotes DR (128). ACE2 regulates bone marrow-derived myeloid angiogenesis, restoring intestinal epithelial and endothelial functions disrupted in DM (129, 130), and alters peptidoglycan biosynthesis by reducing microbiome-associated genes (128).

### 5.5 Affecting metabolites of gut microbiota

SCFAs, composed of acetate, propionate, and butyrate, are small organic metabolites produced by the fermentation of dietary fibers and resistant starch. They have numerous benefits in energy metabolism, intestinal homeostasis, and immune response regulation. It can inhibit the inflammatory response and oxidative stress and affect glycolipid metabolism as a signaling molecule between intestinal flora and the host (110). Glucolipid metabolism disorders and insulin resistance are characteristic manifestations of DR. SCFAs primarily influence glucose and lipid metabolism by regulating the endocrine system. It (especially acetate and butyrate) can specifically stimulate intestinal L-cells and promote the secretion of glucagon-like peptide-1 (GLP-1) and endocrine-regulated peptide (PYY). In obese mice, this improves insulin sensitivity and increases energy expenditure, preventing and treating diet-induced IR (131, 132). In addition, SCFAs reduce IR by inhibiting endotoxin-related inflammation and protecting the intestinal mucosal barrier (133).

## 6 Regulating gut microbiota as a therapeutic strategy for DR treatment

The external environment gradually shapes the diversity of the human intestinal microbiota. Before birth, the fetus is sterile in the intestine and progressively accumulates a specific intestinal flora through exposure to the surrounding environment (134). Childhood and adolescence are critical periods for forming intestinal flora, and individual-specific intestinal flora is formed in adulthood (135). Although the intestinal flora is relatively stable in adulthood, it is also modifiable, with its composition changing with age, diet, lifestyle, and environmental exposure (134). When the gut microbiota in patients with DM and DR is dysregulated, dietary modifications (e.g., probiotic/prebiotic supplementation and low-sugar diet) and fecal transplantation can maintain intestinal homeostasis and improve the condition (136). Moreover, studies on new technologies, such as gut flora editing and synthesis of the gut microbiome to regulate and synthesize gut flora, have been reported (137), providing ideas for using gut flora in treating DR.

Genetic factors have a limited impact on the composition of the host gut microbiota. For example, diets can influence gut microbes in healthy individuals. A high-fat diet is associated with an increased abundance of *Bacteroides*, whereas a high fiber intake is associated with an increased abundance of *Prevotella* (138). The diet also influences the production of intestinal flora metabolites, such as SCFAs, LPS, bile acids, and branched-chain amino acids (BCAA; valine, leucine, and isoleucine) (122). Beli et al. (42) reported that intermittent fasting (IF) can reduce retinal complications (DR) in diabetic mice. In particular, IF can reduce intestinal permeability and promote the production of *tauroursodeoxycholic acid* (TUDCA), a potent activator of Takeda-G-protein-receptor-5 (TGR5) in the retinal ganglion cell layer and can act as a neuroprotective agent. IF also improves intestinal vascular barrier function and lowers plasma peptidoglycan levels. Peptidogly can activates TLR2-mediated signaling cascades and exacerbates DR by interfering with the integrity of retinal endothelial cell junctions (42). In conclusion, these findings reveal that remodeling the intestinal microbiome has a protective effect on the retina, preventing the development of DR. In addition, it has been suggested that the potential mechanism by which DR does not occur in diabetic patients is closely related to intestinal microbiota imbalance, which varies between individuals (139).

## 7 Conclusion and future perspectives

The gut-retina axis concept was developed in response to the dysregulation of gut microbiota observed in patients with retinal diseases such as AMD, DR, and glaucoma. Researchers used antibiotics, probiotics, and diet to reshape the gut microbiota, and the results improved eye disease, providing the link between the gut microbiota and the retina. Subsequent studies revealed further crosstalk between the eye and the gut.

The specificity of the abundance and function of microorganisms and their metabolites in retinal diseases is slowly being elucidated. Scientists have made several advances in the enumeration, characterization, and classification of the human microbiota since the advent of high-throughput sequencing and culture group technologies (134). The most commonly used method for determining microbiome composition was 16S rRNA gene sequencing, which had many limitations for strain-level identification and classification of microorganisms (140). The integrated application of multi-omics, such as macro-genomics, macro-proteomics, and macro-metabolomics, can provide a more accurate and direct interpretation of the functional properties of the intestinal flora for a more accurate understanding of the human micro-ecosystem (141). However, due to individual heterogeneity and the limitations of current diagnostic techniques, interventions on the gut microbiota for disease treatment still be carefully considered. Meanwhile, studies on gut microecology and DR have been reported infrequently compared to other disciplines, and more high-quality studies are required to support this in the future.

The link between microbiota and DR is now well established, and identifying pathogenic or protective microbes is an important step to follow in future. In conclusion, the concept of a gut-retina axis driven by various pathways is being actively investigated, and available data in animals and humans suggest possible therapeutic applications for disease through targeted manipulation of the microbiome.

## Author contributions

HZ reviewed the literature and drafted this review. YM reviewed the literature, gave critical comments, and revised the manuscript. All authors contributed to the article and approved the submitted version.
